# Virtual Service Delivery in Mental Health and Substance Use Care: A Systematic Review of Preference Elicitation Studies

**DOI:** 10.1007/s10597-024-01350-y

**Published:** 2024-09-13

**Authors:** Carly Mallise, Laura Wall, Francesco Paolucci, Kate Davies, Gina La Hera Fuentes, Jessica Wilson, Campbell Tickner, Frances Kay-Lambkin, Milena Heinsch

**Affiliations:** 1 Population Health, Hunter New England Local Health District, Wallsend, NSW 2287 Australia; 2 School of Medicine and Public Health, University of Newcastle, Callaghan, NSW 2308 Australia; 3 School of Psychological Sciences, University of Newcastle, Callaghan, NSW 2308 Australia; 4 Newcastle Business School, University of Newcastle, Callaghan, NSW 2308 Australia; 5 Department of Sociology and Business Law, University of Bologna, Bologna, BO 40126 Italy; 6 Homelessness NSW, Woolloomooloo, NSW 2011 Australia; 7 School of Humanities, Creative Industries and Social Sciences, University of Newcastle, Callaghan, NSW 2308 Australia; 8 Brain and Mind, Murdoch Children’s Research Institute, Parkville, VIC 3052 Australia; 9 School of Social Work, University of Tasmania, Hobart, TAS 7005 Australia; 10 Hunter Medical Research Institute, New Lambton Heights, NSW 2305 Australia; 11 Hunter New England Population Health, Longworth Avenue, Wallsend, NSW 2287 Australia

**Keywords:** Telehealth, Preferences, Discrete choice experiment, Service user, Service provider, Health services

## Abstract

Mental health and substance use disorders affect the lives of many people worldwide. Prevention and treatment of these conditions is important for optimal health and wellbeing, yet service access barriers are common. Virtual models of care may help to reduce barriers to receiving care. However, to facilitate uptake and use of virtual services, they need to appeal to patients and clinicians. This systematic review aimed to synthesise preference elicitation studies to determine what features of virtual mental health and substance use care are preferred by service users and service providers. Following the PRISMA guidelines for systematic reviews, we searched PubMed, PsycINFO, EconLit, MEDLINE, CINAHL, Academic Search Ultimate, and ProQuest Central for all available studies from database inception until May 2023. The Mixed Methods Appraisal Tool was used to assess the methodological quality of included studies. Nineteen studies met the eligibility criteria. However, none examined preferences for elements of different models of virtual care. Across the included studies, we identified 41 unique features that mapped to four themes of mental health and substance use care (‘service’, ‘treatment’, ‘clinician’ and ‘additional supports’). Participant preferences were for individual, in-person, effective, flexible, and low-cost treatment. These preferences varied based on demographic factors, such as culture, gender, and participant type (e.g., patients, clinicians, general population). A user-centred approach should be adopted when designing and implementing mental health and substance use services. While preferences for features of mental health and substance use services more broadly are known, preferences for different models of virtual care remain unexplored. Future research should examine what features of virtual services would lead to optimal uptake and use across different users and stakeholders.

## Introduction

Mental health and substance use disorders are common health conditions that affect the lives of countless people worldwide. In 2018, it was estimated that 20% of the Australian population had a mental health or substance use disorder (Australian Bureau of Statistics, 2018), with rates of 13.2–16.7% reported overseas (Baker, 2020; Jha et al., 2019; Stagnaro et al., 2018). Mental health and substance use disorders represent 5% of disease burden and account for around 14.3% of deaths globally (Dattani et al., 2021; Walker et al., 2015). For individuals with severe mental illness, adverse outcomes such as poorer physical health, stigma, and homelessness commonly co-occur (Dubreucq et al., 2021; Elbogen et al., 2021; Robson & Gray, 2007). This highlights the importance of accessible and multi-faceted services to ensure that people can receive the care they need. The uptake and use of mental health and substance use treatment has been found to help individuals manage symptoms of their condition(s), improving their physical and social wellbeing (Clark et al., 2018; Nakao et al., 2021). Furthermore, the sooner one access care, the less their mental health status declines (Reichert & Jacobs, 2018; Smith et al., 2018). Although effective treatments for mental health conditions exist, delays in accessing mental health care frequently occur and many affected people remain untreated (Bidargaddi et al., 2020). Thus, accessible mental health treatment and care is critical.

In recent years, virtual care delivery, which encompasses the use of e-health, m-health, and telehealth, has surged across all healthcare services due to the COVID-19 pandemic, particularly in mental health and substance use services (Ellis et al., 2021; Sorkin et al., 2021; van Kessel et al., 2022; Zhu et al., 2021). Virtual care addresses some of the barriers in accessing traditional in-person mental health and substance use services, such as poor affordability and inaccessibility (Coombs et al., 2021; Moroz et al., 2020), with telehealth of particular interest as it provides the closest approximation to in-person care. For certain mental health conditions, virtual care offers the same effectiveness of care as traditional in-person consultation (Greenwood et al., 2022; Krzyzaniak et al., 2021; Scott et al., 2022a; Scott, Clark, Scott et al., 2022a, b), but more convenience as patients can receive care from their own homes, reducing the need to travel (Berardi et al., 2024; Polinski et al., 2016). This is particularly beneficial for patients living in rural and remote areas, who might find it burdensome to travel long distances to receive treatment (Butzner & Cuffee, 2021). Studies have also shown that virtual care can reduce the wait times for outpatient services (Caffery et al., 2016; Mahmoud et al., 2021; Uscher-Pines et al., 2020; Valentine et al., 2021). Additionally, the option to seek care from home can mitigate the stigma associated with accessing mental health treatments (Kim et al., 2022; Kim & Tesmer, 2021; Philip et al., 2022).

Despite its many benefits, the implementation of virtual care does present unique challenges. Common barriers to virtual care use include a lack of access to technology, difficulties establishing strong client-patient rapport, and concerns about privacy (Hughto et al., 2021; Naal et al., 2021). To ensure that people can access the care they need, especially when virtual care is the only option, it is critical that this service delivery modality is implemented in a way that promotes effective, sustainable, and equitable uptake. One way of addressing this need is to employ a person-centred approach by identifying the features of virtual care delivery that are important to service users and service providers and most likely to influence their utilisation of a service. As a first step, it is important to identify the factors that contribute to people using (or not using) mental health and substance use services that involve an element of virtual care. As a second step, it is important to understand the trade-offs that people make between these factors (e.g., between wait time and type of provider), and the extent to which each factor leads to greater (or lesser) use of a service for different groups of people. This second step allows analyses that can predict the change in uptake for a service, with a change in a factor (e.g., how many more clients would use the service if the wait time was reduced by half) providing clear, easily implementable recommendations to services to ensure effective, sustainable, and equitable uptake.

These steps are best achieved through preference elicitation methodologies, such as discrete choice experiments (DCEs), which use a systematic process to directly ask individuals for their preferences on a product or service. Compared to qualitative methodologies, preference elicitation methods can produce more precise preference data as individuals are explicitly communicating their likes, dislikes, and priorities, within realistic scenarios, comparable across all respondents (Soekhai et al., 2019). As such, they are ideal methods for empirically determining which factors are most important to service users and providers, and therefore likely to influence their utilisation of the services (Clark et al., 2014; Ryan et al., 2008). In a DCE, participants are presented with a series of realistic hypothetical products or services (e.g., a health service), which are comprised of key *attributes* (e.g., waiting time) that are believed to influence utilisation of the product/service. The expression of these attributes differs between the hypothetical products/services through pre-defined *levels*, such as one, two or three weeks (for waiting time). Participants indicate which product/service they prefer and/or would use. Their choices, paired with the attribute levels presented to them, provide information on the ‘ideal’ product/service. An evidence-based approach, often using systematic reviews and qualitative methods, is utilised to select the attributes and levels included in a DCE or other preference elicitation study (Ryan et al., 2008). A synthesis of the features (i.e., attributes and levels) included in preference elicitation studies, is thus an efficient method of exploring what important factors have been found, and what gaps in knowledge remain. This systematic review aimed to determine what factors have been found to influence service user and service provider preferences for virtual service delivery in mental health and substance use care.

## Methods

The research team used Covidence (Veritas Health Innovation, 2021) to conduct screening, full-text review, quality assessment and data extraction. EndNote (The EndNote Team, 2013) was used to import exported references from the databases and the manual search into Covidence. A review protocol was created and made available to the public using the Open Science Framework on September 29th, 2021 (DOI: 10.17605/OSF.IO/ZBG3J).

### Search Strategy

A systematic search was conducted across seven health, economics, and social science databases: PubMed, PsycINFO, EconLit, MEDLINE, CINAHL, Academic Search Ultimate, and ProQuest Central (Consumer Health, Health & Medical Collection, Nursing & Allied Health, Psychology, Public Health & Social Science databases). The search was conducted by one independent reviewer (CM) for all articles published from database inception until May 23rd 2023, using search terms related to preference elicitation of service modality in mental health and substance use populations (Table 1). Additionally, a manual search was conducted by four independent reviewers (CM, JW, KD, LW) of the reference lists of relevant excluded review articles and included articles, as well as relevant excluded protocol articles and dissertations, to identify relevant peer-reviewed original research articles.

**Table 1:** Database search terms

		AND	AND	AND	AND
OR	mental	service*	preference*	telehealth	discrete choice experiment
OR	psycholog*	centre*	attitude*	ehealth	discrete-choice experiment
OR	psychiatr*	center*	barrier*	e-health	discrete-choice conjoint experiment
OR	mind	clinic*	enabler*	mhealth	conjoint analysis
OR	alcohol	practice*	facilitator*	m-health	multi-criteria decision analysis
OR	drug*	program*	view*	(modality or mode or delivery) adj5 online	multi-criteria decision method
OR	substance*	healthcare provider	belief*	(modality or mode or delivery) adj5 digital	
OR	smoking		value*	(modality or mode or delivery) adj5 virtual	
OR	addiction		perception*	(app* or technolog* or text or sms or email or telephone or teleconferenc* or video conferenc*) adj5 health	
OR			skeptic*		
OR			sceptic*		
OR			behavioural intention		
OR			behavioral intention		
OR			opinion*		
OR			perspective*		
OR			appraisal		

Note *Indicates a wildcard which means that any term stemming from the base is searched (e.g., psycholog* captured psychologist, psychologists, psychology, psychological). adj5 is a search method that requires the terms in parentheses to be within 5 words of the subsequent term

### Eligibility Criteria

Titles and abstracts of articles were screened for relevance by two independent reviewers (CM, FKL, KD or LW), with conflicts resolved either via consensus or a third reviewer (FKL). Articles that were screened as being relevant moved to the full-text review stage where they were reviewed against eligibility criteria by CM and one other independent reviewer (CT, GLHF or KD), with conflicts resolved by a third independent reviewer (LW). Articles were included if they were (i) written in English, (ii) reported on peer-reviewed original research, and (iii) used a preference elicitation measure (e.g., discrete choice experiment) for virtual service delivery in either (a) mental health or (b) substance use service settings. Articles were excluded if they (i) were written in a language other than English, (ii) were a review article, conference paper, book, dissertation, grey literature, or not peer-reviewed, (iii) used non-human participants, (iv) were not focused on either (a) mental health or (b) substance use services, v) did not use a preference elicitation measure, vi) did not include a virtual care modality, or vii) did not report on service delivery features.

### Quality Assessment and Data Extraction

The *Mixed Methods Appraisal Tool* (version 2018; Hong, 2018; Pluye et al., 2009) was used by two reviewers (CM, LW) to appraise the quality of the studies included in the systematic review. We completed the two screening questions applicable to all study designs and the five questions specific to the ‘quantitative descriptive’ study design, using the tool’s yes/no/can’t tell response options. Discrepancies in the quality appraisal of the two reviewers were discussed until consensus was achieved. No studies were excluded based on quality; however the quality of the studies was considered when interpreting the results.

One reviewer (CM) independently extracted the following data from the included articles: country, setting (e.g., hospital), service type (i.e., mental health or substance use), sample size, participant type (e.g., service user or provider), participant demographics (e.g., age, gender), preference elicitation tool (e.g., DCE or rating scale), and study outcomes. For quality assurance purposes, 20% of the extracted data was checked by another reviewer (LW). A meta-analysis was not feasible due to heterogeneity in the attributes and levels of the studies included. Instead, results are presented as a qualitative synthesis.

## Results

**Fig. 1:**
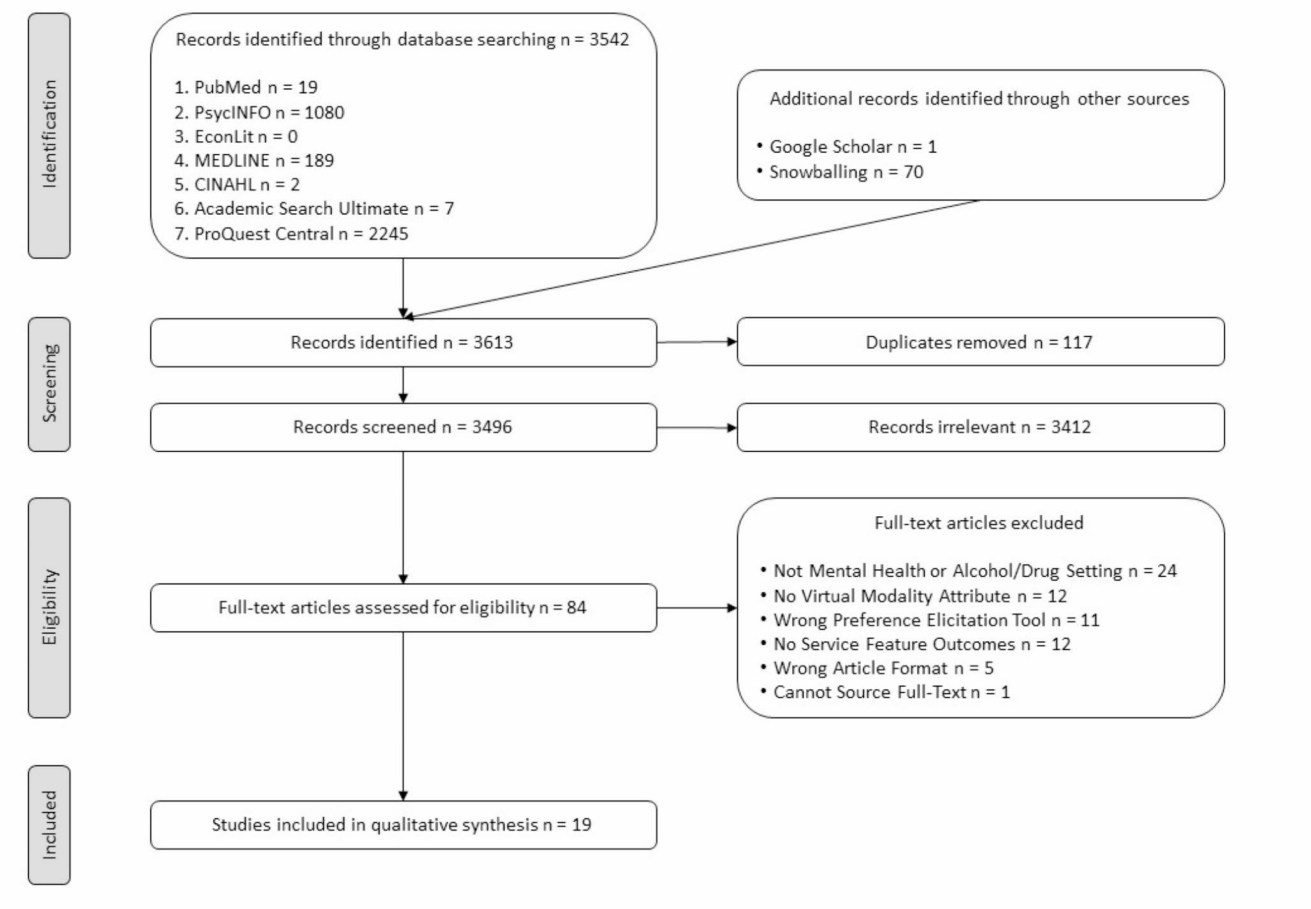
PRISMA flowchart of the review process

While we sought to synthesise preferences for features of virtual mental health and substance use service delivery in this review, we did not identify any studies that directly investigated this. However, 19 studies were identified by our search strategy that met our eligibility criteria, which are subsequently included in this review. We included these studies because they examined preferences related to some component of virtual care. Additionally, these studies provide evidence on what features are generally important to service users and providers when making decisions about their care and the services they access. As such, the following sections mainly report on preferences for aspects of mental health and substance use treatment services other than virtual care. Figure 1 depicts the Preferred Reporting Items for Systematic Reviews and Meta-Analyses (PRISMA) flowchart detailing the number of articles at each stage of the review.

### Study Characteristics

Table 2 presents the study characteristics and participant characteristics. The 19 studies were conducted by 12 research teams. Most studies were published in 2010 or after (95%), were conducted in North America (63%), and elicited preferences for solely mental health service features (74%) using DCEs/conjoint analysis (90%). Sample sizes ranged from 42 to 1984 participants (mean = 374, median = 218). Seven of the 19 studies used more than one recruitment method, with face-to-face (32%) and online (26%) being the most frequently employed. Participants were primarily women in most studies (*n* = 14), with only one study focusing solely on men (Dwight-Johnson et al., 2013). Mean age ranged from 35 to 59 years with a large spread as most participants were in their early 20’s to mid-30’s. Most participants had undertaken or attained more than a high school degree (61-100%) in 12 of the 19 studies. Of the studies that reported on language (*n* = 8), English was most frequently reported as preferred/first language (71-91%), but two studies had Spanish-speaking participants as the majority (Dwight-Johnson et al., 2004, 2010). Three studies (Hawke et al., 2021a, b; Klein & Cook, 2010) reported on residential location, with most participants residing in a metropolitan area (68-79%) compared to regional and rural.

**Table 2:** Study characteristics of included articles

Author year, country	Preference elicitation tool	Recruitment method	Sample size	Participant demographics	Mental health/substance use focus and treatment history
Dwight-Johnson 2004, United States	Conjoint Analysis	Face-to-Face	42	Patients 81% 31–65 years 95% women 52% <6 years of education 93% Latino/Latina 94% born in Mexico 86% Spanish (primary language)	Major depressive disorder/dysthymia 29% past counselling 33% past psychotropic medication 5% currently in treatment
Dwight-Johnson 2010, United States	Conjoint Analysis	Face-to-Face	339	Patients 49.8 ± 12.6 (mean) years 84% female 56% <6 years of education 20% 6–11 years of education 24% ≥ high school graduate 100% Latino/Latina 44% born in Mexico 27% born in El Salvador 10% born in United States 19% born elsewhere 75% Spanish (primary language)	Major depressive disorder/dysthymia 26% past counselling 71% past medication
Klein, 2010, Australia	Rating Scale	Online, poster/flyer	218	General population, university students 36.57 ± 14.5 (mean) years 76% female 26% ≤ completed secondary education 57% undertaking/completed undergraduate education 17% undertaking/completed postgraduate education 79% born in Australia 9% rural location 15% regional location 76% metropolitan location	Mental health (unspecified) 73% general practitioner 84% psychologist 42% psychiatrist 57% counsellor 48% self-help book 71% information website 6% online counselling 5% Internet-based program with therapist-assistance 12% internet-based program without therapist-assistance 29% telephone counselling service 63% past medication
Lau 2012, Canada	Discrete Choice Experiment	Email, newsletter, oster/flyer	151	Health authority employees 43.8 ± 10.81 (mean) years 89% female 39% < university degree 61% university degree	Depressive symptoms 11% past mindfulness-based cognitive therapy
Dwight-Johnson 2013, United States	Conjoint Analysis	Face-to-Face	63	Patients 49% 60–64 years 100% men 71% ≥ high school graduate 84% English (preferred language)	Major depressive disorder 63% depression treatment in past year
Becker 2016, Canada	Discrete Choice Experiment	Face-to-Face, email	562	Patients, family members, service providers 7% 16–20 years 27% 21–35 years 51% 36–55 years 16% >55 years 71% female 71% ≤ high school education 81% > high school education 83% born in Canada 89% English (preferred/only language)	Mental health (unspecified)
Batterham, 2017, Australia	Rating Scale	Online	438	General population 34.9 ± 15.5 (mean) years 79% female 37% < post-secondary education 14% certificate, diploma, or associate degree 24% bachelor’s degree 23% higher degree 89% English (language spoken at home)	Anxiety symptoms/ suicidal ideation
Cunningham 2017, Canada	Discrete Choice Experiment	Not reported	909	University students 92% 16–20 years 8% 21 + years 75% female 86% 1st year university student 14% ≥ 2nd year university student 71% English (preferred/only language) 71% born in Canada	Mental health (unspecified) 84% not currently using or looking for mental health service 9% currently looking for mental health service 7% currently using mental health service
Becker 2019, Canada	Discrete Choice Experiment	Face-to-Face, email	516	Patients, family members, service providers 4% 16–20 years 27% 21–35 years 54% 36–55 years 15% >55 years 73% female 17% ≤ high school education 83% > high school education 89% English (preferred/only language) 85% born in Canada	Mental health (unspecified)
Lokkerbol 2019a, Netherlands	Discrete Choice Experiment	Online	165	Patients 41% 18–24 years 16% 25–30 years 16% 31–40 years 10% 41–50 years 13% 51–60 years 4% 61 + years 90% female 4% low education level 32% middle education level 64% high education level	Depressive disorders 84% any past treatment 59% past medication
Lokkerbol 2019b, Netherlands	Discrete Choice Experiment	Online	126	Patients 39% 18–24 years 20% 25–30 years 14% 31–40 years 11% 41–50 years 15% 51–60 years 2% 61 + years 90% female 5% lower occupational 33% higher occupational 62% academic	Anxiety disorders 72% any past treatment 52% past medication
Muntingh 2019, Netherlands	Discrete Choice Experiment	Phone, mail	109	Patients 41.3 ± 12.7 (mean) years 64% female 15% ≤ 13 years of education 33% 13–14 years of education 52% ≥ 15 years of education	Anxiety disorder or depressive disorder 19% experience with self-help 6% experience with e-health
Katz 2020, United States	Discrete Choice Experiment	Phone, mail	61	Patients 58.5 ± 11.0 (mean) years 87% male 13 years of education (median) 83% white	Tobacco smoking 32% past counselling 86% past medication
Phillips 2021, Germany	Discrete Choice Experiment	Market research agency	1984	General population 51.2 ± 13.2 (mean) years 58% female 57% high school diploma (university entry unqualified) 21% high school diploma (university entry qualified) 22% university degree	Mental health (unspecified) 62% past counselling 7% past online therapy app use
Bastien 2021, Canada	Discrete Choice Experiment	Face-to-Face, poster/flyer, word of mouth	165	Patients 41.2 ± 10.4 (mean) years 70% men 36% < high school graduate 64% ≥ high school graduate 86% white	Depressive symptoms (in people with opioid use disorder) 100% current opioid agonist treatment 33% current antidepressants 15% current counselling 10% waiting to receive treatment 55% not currently receiving or waiting for treatment
Hawke 2021a, Canada	Discrete Choice Experiment	Email	388	Service providers 27% 18–29 years 35% 30–39 years 20% 40–49 years 17% 50 + years 85% female 63% ≤ bachelor’s degree 37% postgraduate degree 20% rural location 21% medium urban location 59% large urban location	Mental health and/or substance use (unspecified)
Hawke 2021b, Canada	Discrete Choice Experiment	Poster/flyer	274	Family members Age not reported 91% women 5% ≤ high school graduate 15% some university 80% university graduate 85% white 91% English (first language) 85% born in Canada 15% rural location 18% medium urban location 67% large urban location	Mental health and/or substance use (unspecified)
Phillips 2022, Germany	Discrete Choice Experiment	Market research agency	200	Service providers 48 years (mean) 57% male Education not reported	Mental health (unspecified)
Tauscher 2023, United States	Discrete Choice Experiment	Online	400	Patients 32 years (median) 63% male 13% high school diploma 61% undergraduate degree 26% postgraduate degree 73% white	Alcohol or other substance use disorder symptoms

Abbreviations N/A = not applicable. Note Katz 2020 reported on two DCEs; the one that met our inclusion criteria is included here. We listed the following participant demographics, if reported by the included article: population (patient, family member or service provider), age, gender, education, race, language, country of birth, and location type. The terminology used by the included studies when referring to participant gender is reported here

Most of the studies (*n* = 17) included participants that were service users; people with a mental health or substance use condition (*n* = 11), carers/family members (*n* = 3), university students (*n* = 2), people from the general population (*n* = 3), and health authority employees (*n* = 1). Service users who were people with a mental health or substance use condition, and their carers/family members, were drawn from various settings including outpatient mental health clinics, primary care, and mental health organisations’ social media channels. Only four studies included participants that were service providers (clinicians and/or service administrators). Service provider background was not provided in three of the four studies. However, the one that did report on professional background had a sample comprised mainly of psychotherapists/psychiatrists. Regarding the mental health/substance use focus, depression was the most common across the studies (*n* = 7). Other focuses included anxiety (*n* = 2), tobacco smoking (*n* = 1), and opioid use disorder (*n* = 1). Ten studies did not focus on a particular mental health and/or substance use disorder. Of the studies reporting on treatment history (*n* = 9), most service user participants (62-100%) were either currently being treated or had previously been treated for their mental health or substance use conditions.

### Quality Assessment of Included Studies

Table 3 reports the percentage of MMAT (Hong, 2018; Pluye et al., 2009) items that met methodological requirements within each article after consensus. Agreement between the two reviewers (CM, LW) was very good, with agreement percentages resulting as either 71% (9 articles), 86% (7 articles), or 100% (3 articles) for the 19 included articles. All 19 articles included clear research questions and reported on data that addressed those questions. Of the 19 articles, two (Lau et al., 2012; Phillips et al., 2021) had excellent methodological quality with 100% of items meeting requirements. Some methodological constraints, however, were identified in 17 of the articles. The most common limitations of articles were not demonstrating a sample representative of the target population (80% failed to meet item) and not having low risk of non-response bias (80% failed to meet item). This was often due to the samples being over representative of females and highly educated people, low response rates, and not reporting analyses comparing the demographics of people who did and did not participate. Despite these concerns, the quality of the studies overall was very good, with 14 of the 19 articles having relevant sampling strategies, appropriate measurements, and appropriate statistical analyses.

**Table 3:** Results on the mixed methods appraisal tool assessing the quality of the included articles

Article	S1	S2	4.1	4.2	4.3	4.4	4.5.	
	Are there clear research questions?	Do the collected data allow to address the research questions?	Is the sampling strategy relevant to address the research question?	Is the sample representative of the target population?	Are the measurements appropriate?	Is the risk of nonresponse bias low?	Is the statistical analysis appropriate to answer the research question?	% ‘Yes’
Dwight-Johnson 2004	Yes	Yes	Yes	No	Yes	No	No	57
Dwight-Johnson 2010	Yes	Yes	Yes	No	Yes	Can’t Tell	Yes	71
Klein, 2010	Yes	Yes	Yes	No	Can’t Tell	Can’t Tell	Yes	57
Lau 2012	Yes	Yes	Yes	Yes	Yes	Yes	Yes	100
Dwight-Johnson 2013	Yes	Yes	Yes	No	Yes	No	Yes	71
Becker 2016	Yes	Yes	Yes	Can’t Tell	Yes	Can’t Tell	Yes	71
Batterham, 2017	Yes	Yes	Yes	No	Yes	No	Yes	71
Cunningham 2017	Yes	Yes	No	No	Yes	No	Yes	57
Becker 2019	Yes	Yes	Yes	Can’t Tell	Yes	No	Yes	71
Lokkerbol 2019a	Yes	Yes	No	No	Yes	No	Yes	57
Lokkerbol 2019b	Yes	Yes	No	No	Yes	No	Yes	57
Muntingh 2019	Yes	Yes	Yes	Yes	Yes	No	Yes	86
Katz 2020	Yes	Yes	Yes	Can’t Tell	Yes	No	Yes	71
Phillips 2021	Yes	Yes	Yes	Yes	Yes	Yes	Yes	100
Bastien 2021	Yes	Yes	Yes	Can’t Tell	Yes	Can’t Tell	Yes	71
Hawke 2021a	Yes	Yes	Yes	Can’t Tell	Yes	Can’t Tell	Yes	71
Hawke 2021b	Yes	Yes	Yes	Can’t Tell	Yes	Can’t Tell	Yes	71
Phillips 2022	Yes	Yes	Yes	Can’t Tell	Yes	Can’t Tell	Yes	71
Tauscher 2023	Yes	Yes	Yes	Can’t Tell	Yes	Yes	Yes	86
% ‘Yes’	100	100	84	16	95	16	95	

### Study Outcomes: Preferences for Characteristics of Virtual Service Delivery for Mental Health and Drug Care

**Fig. 2:**
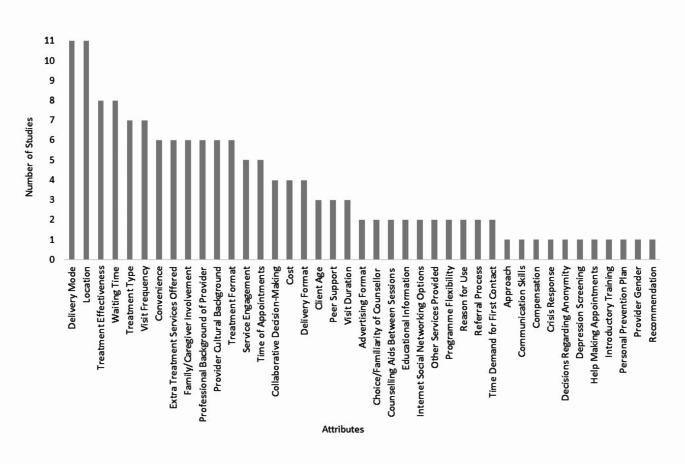
A stacked column chart showing the number of studies that included each of the 41 unique attributes

Seventeen studies employed DCEs, and two studies used rating surveys. The attributes from the DCEs and questions from the rating surveys were grouped thematically to synthesise the mental health and substance use care features included in the studies. After thematic synthesis, 41 unique features were identified for mental health and substance use care across all the studies (Fig. 2). Features were themed into one of four overarching aspects of mental health and substance use care to aid in synthesis: ‘service features’, ‘treatment features’, ‘clinician features’ and ‘additional supports’. The following sections describe the statistically significant preferences of features that were included in four or more studies, as features identified in fewer studies did not provide sufficient evidence to synthesise. Preferences of features were not presented separately for service users and service providers due to the small number of studies in the latter group. However, where relevant, the participant group is noted in the presentation of findings. All features identified in each theme and their associated preferred option can be found in Table 4.

**Table 4:** Preferences for features included in the studies

Theme and features	Study	Preferences
Service Features		
Advertising Format	6	Conventional LC: Public awareness events in the community Convenient LC: Television and radio
	8	Psych LC: This service is advertised at public awareness events on campus Alternative LC: This service is advertised on university Internet sites like Mac Connect Hesitant LC: ns
Client Age	6	Conventional LC: This service feels like it is for people ages 18 and older Convenient LC: This service feels like it is for people ages 12 and older
	16	Services for ages 12–29, in a youth-only setting
	17	Comprehensive, Integrative Service Access LC: Services for ages 12–29, in a youth-only setting Service Process Feature LC: Services for ages 12–29, in a setting that also has services for adults 29+ Caregiver Involvement LC: Services for ages 12–24, in a youth-only setting
Compensation	18	Time + lump sum
Convenience	1	Telephone appointments and bus pass
	2	Telephone appointments and bus pass
	5	White non-Hispanic: Telephone appointments Mexican origin: Telephone appointments and bus pass
	13	Scheduled face-to-face counseling in VA clinic
	16	E-health services are offered 24/7 alongside in-person services during office hours
	17	Comprehensive, Integrative Service Access and Caregiver Involvement LCs: E-health services are offered alongside in-person services Service Process Feature LC: Can schedule appointments (via e-health services)
Cost	2	Lower^^^
	5	White non-Hispanic & Mexican origin: Lower^^^
	14	€0
	19	Lower^^^
Decisions Regarding Anonymity	6	Conventional & Convenient LC: If they want, people give their name when contacting this service
Delivery Format	3	Information website
	7	Text (information)
	12	ns
	14	Video
Delivery Mode	3	Total & e-preferers: Internet-based program with therapist-assistance Non e-preferers: Telephone (counselling)
	4	Total, LC 1 & LC 3: Face-to-face LC 2: Tech-enabled LC 4: ns
	6	Conventional & Convenient LC: Face-to-face
	7	Laptop/desktop computer
	8	Psych & Alternative LC: Choice of phone, Internet, or face-to-face Hesitant LC: Face-to-face
	10	Total: ns All Subgroups: Combination
	11	Total: ns All Subgroups: Combination
	14	Face-to-face
	15	Live therapist
	18	80% face-to-face and 20% online
	19	Face-to-face meetings
Extra Treatment Services Offered	5	White non-Hispanic & Mexican origin: Insomnia
	6	Conventional & Convenient LC: Gives information about psychological treatments
	7	Strategies to change unhelpful thoughts and negative feelings
	9	Professional & Patient LC: All patients get help with alcohol or drug problems
	16	Mental health and substance misuse counseling, medication management, and physical/sexual health
	17	All three LCs: Mental health and substance misuse counselling, medication management, and physical/sexual health
Introductory Training	14	Phone
Location	1	ns
	2	Primary care
	5	White non-Hispanic: ns Mexican Origin: Home
	6	Conventional LC: Clinic or hospital Convenient LC: Home
	7	Home
	8	Psych & Alternative LC: campus student health centre Hesitant LC: Not ‘Home’
	9	Professional & Patient LC: Office in the community
	15	At an opioid agonist treatment clinic
	16	Youth café and recreation centre
	17	Comprehensive, Integrative Service Access and Service Process Feature LCs: Office that specializes in mental health services Caregiver Involvement LC: Youth café and recreation centre
	19	Community office (e.g., medical office or counseling center)
Referral Process	5	Conventional LC: ns Convenient LC: People can refer themselves
	8	Psych & Alternative LC: Students can refer themselves Hesitant LC: Students must be referred by a family doctor
Service Engagement	6	Once a week this service educates the community about mental health
	8	Psych LC: Once a week this service educates the community about mental health Alternative & Hesitant LC: Once a month this service educates the community about mental health
	9	Professional & Patient LC: People who have experienced mental health problems helped design this service
	16	Youth and caregivers are on an advisory group that gives feedback on services and evaluation
	17	All three LCs: Youth and caregivers are on an advisory group that gives feedback on services and evaluation.
Time Demand for First Contact	6	Conventional & Convenient LC: First contact lasts 1 h
	8	Psych & Alternative LC: First contact lasts 1 h Hesitant LC: ns
Time of Appointments	4	Total, LC 1 & LC 3: ns LC 2: Employer’s time LC 4: Own time
	6	Conventional LC: Appointments at a convenient time for both patients and the service Convenient LC: No appointments needed; can be used anytime
	9	Professional LC: Appointments are on weekday afternoons Patient LC: Appointments are on weekday evenings
	16	Monday to Friday, 9 AM-9 PM, and Saturday, 9 AM-5 PM
	17	Comprehensive, Integrative Service Access and Caregiver Involvement LCs: 24/7 Service Process Feature LC: Monday to Friday, 9AM-9PM, and Saturday, 9AM-5PM
Visit Duration	7	Five 60-minute sessions over 5 weeks
	12	½ hour per week
	15	40 min
Visit Frequency	5	White non-Hispanic & Mexican origin: ns
	10	Total & Lower Impairment subgroup: ns All other subgroups: Weekly
	11	Low Age: Weekly High education: Fortnightly Total & all other subgroups: ns
	12	Once every 3 months
	13	5 or more follow-up sessions
	15	For 2 months
	19	One time a week
Waiting Time	4	Total & LC 1: One Week All other LC: ns
	6	Conventional & Convenient LC: Immediately
	8	Psych LC & Alternative LC: Immediately Hesitant LC: ns
	10	Total: ns All subgroups: Less^^^
	11	Total: ns All subgroups: Less^^^
	16	See a counselor for the first time immediately, during office hours
	17	All three LCs: Immediately, during office hours
	19	ns
Treatment Features		
Approach	7	Information about mental health problems
Collaborative Decision-Making	9	Professional & Patient LC: Patients and clinicians together choose the treatment
	13	Emphasizes that it is your choice on when and how to quit
	16	Youth and service provider work together to decide what personal information to share with caregivers and how that can be helpful
	17	Comprehensive, Integrative Service Access and Service Process Feature LC: Youth and service provider work together to decide what information to share with caregivers Caregiver Involvement LC: Information is available to caregivers, with youth consent
Depression Screening	5	WNH: GP MO: ns
Programme Flexibility	2	Tailored
	12	Choose individual modules or exercises
Treatment Effectiveness	4	Total, LC 1, LC 3 & LC 3: Lower chance of relapse^^^ LC 4: ns
	6	Conventional & Convenient LC: People who have experienced mental health problems say this service is helpful
	8	Psych & Alternative LC: Students who have experienced mental health problems say this service is helpful Hesitant LC: ns
	12	The risk of relapse decreases from 60–36%
	13	22% (quit rate at 1 year)
	14	Yes
	18	More^
	19	Yes
Reason for Use	7	If I had been diagnosed with a mental health problem
	9	Professional & Patient LC: Main goal is to reduce anxiety, depression or psychosis
Recommendation	18	Professional societies
Treatment Format	1	Individual
	2	ns
	4	Total, LC 1 & LC 2: Individual LC 3 & 4: ns
	9	Professional LC: Most sessions alone with clinician, some with a small group of patients and a clinician Patient LC: Individual
	10	Total: ns All subgroups: individual
	11	Total: ns All subgroups: individual
Treatment Type	1	Counselling and medication
	2	Counselling and medication
	3	Total & e-preferers: Internet-based program with therapist-assistance non e- preferers: Prescribed medication
	5	White non-Hispanic: Medication Mexican origin: Counselling
	8	Psych, Alternative & Hesitant LC: Choice of alternative treatment, psychotherapy & med
	9	Professional & Patient LC: Choice of alternative treatment, psychotherapy & med
	12	ns
Clinician Features		
Communication Skills	13	Always listens carefully and explains things clearly
Choice/Familiarity of Counsellor	13	Someone whom you see more than half of the time
	19	You choose from a list
Professional Background of Provider	3	Total, e-preferers & non e-preferers: Psychologist
	5	WNH: ns MO: Psychiatrist
	6	Conventional LC: Psychologist or psychiatrist Convenient LC: Mental health nurse
	8	Psych LC & Hesitant LC: Psychologist or psychiatrist Alternative LC: Peer counsellor who has experienced mental health problems
	9	Professional LC: Mental health nurse Patient LC: Psychologist or psychiatrist
	13	Clinical counsellor (e.g., nurse)
Provider Cultural Background	5	White non-Hispanic & Mexican origin: ns
	6	Conventional LC & Convenient LC: Same culture, if wanted
	8	Psych LC & Alternative LC: Same culture, if wanted Hesitant LC: ns
	9	Professional & Patient LC: Same culture, if wanted
	16	Services are culturally sensitive and trauma informed
	17	Comprehensive, Integrative Service Access and Service Process Feature LCs: Services are culturally-sensitive and trauma-informed Caregiver Involvement LC: Cultural background is not considered when picking a service or service provider
Provider Gender	5	White non-Hispanic & Mexican origin: ns
Additional Supports		
Counselling Aids Between Sessions	9	Professional LC: Includes helpful text messages and phone help Patient LC: Includes phone help
	13	Print materials (e.g., brochure on quitting)
Crisis Response	9	Professional & Patient LC: Patients in crisis can get help 24 h per day
Educational Information	1	Group, written and video
	2	Individual meeting
Family/Caregiver Involvement	2	Yes
	5	White non-Hispanic & Mexican origin: yes
	6	Conventional LC & Convenient LC: The service and people using the service decide if families are involved
	9	Professional LC & Patient LC: Clinicians and patients decide together whether families are involved
	16	Caregivers are involved in family counseling with youth, with youth consent
	17	Comprehensive, Integrative Service Access and Service Process Feature LCs: Caregivers involved in counseling with youth, with consent Caregiver Involvement LC: Caregivers involved in youth counseling decisions, with consent
Help Making Appointments	1	Yes
Internet Social Networking Options	6	Conventional LC: Professionally supervised Internet site where people talk about mental health problems Convenient LC: Internet site where professionals answer questions about mental health problems
	9	Professional & Patient LC: Has an internet site where patients ask professionals
Peer Support	14	Online community plus face-to-face meetings
	16	Youth can be matched to an ongoing trained peer support worker to learn life skills and help them with services they need
	17	Comprehensive, Integrative Service Access and Service Process Feature LCs: Youth can be matched to an ongoing trained peer support worker to learn life skills and help them with services they need Caregiver Involvement LC: Recreational activities led by trained peer support worker
Personal Prevention Plan	12	Included in intervention
Other Services Provided	16	Choice of education, employment, housing, income support, and legal support services
	17	All three LCs: Choice of education, employment, housing, income support, and legal support services

Note: The subgroup analyses for Lokkerbol 2019a and 2019b were (a) age (low vs. high), (b) education (low vs. high), and impairment (low vs. high). Katz 2020 reported on two DCEs; the one that met our inclusion criteria is included here. Delivery mode, location, and visit frequency levels were combined in one attribute in Tauscher 2023. Abbreviations: LC = Latent Class

^^^ Included in analyses as a continuous variable

^1^ Dwight-Johnson 2004

^2^ Dwight-Johnson 2010

^3^ Klein, 2010

^4^ Lau 2012

^5^ Dwight-Johnson 2013

^6^ Becker 2016

^7^ Batterham, 2017

^8^ Cunningham 2017

^9^ Becker 2019

^10^ Lokkerbol 2019a

^11^ Lokkerbol 2019b

^12^ Muntingh 2019

^13^ Katz 2020

^14^ Phillips 2021

^15^ Bastien 2021

^16^ Hawke 2021a

^17^ Hawke 2021b

^18^ Phillips 2022

^19^ Tauscher 2023

### Service Feature Preferences

Eighteen features were identified that aligned with the theme of service features. All studies included at least one of the features in this theme, with 10 features included in four or more studies.

Service Location: Preferences varied regarding the location of service delivery, with some studies reporting a preference for clinical settings (Bastien et al., 2021; Becker et al., 2016; Cunningham et al., 2017; Dwight-Johnson et al., 2010; Hawke et al., 2021b; Tauscher et al., 2023) and others reporting a preference for the home environment (Batterham & Calear, 2017; Becker et al., 2016; Cunningham et al., 2017; Dwight-Johnson et al., 2013). Becker et al. (2019) found that participants preferred community-based offices, over options such as their home, family doctor’s office or hospital. Demographic factors influenced location preferences in some studies. In a United States (US) study, Dwight-Johnson et al. (2013) found that men of Mexican origin preferred their home whereas White non-Hispanic men had no significant preference. Becker et al. (2016) reported that participants who were predicted to access conventional services (i.e., mostly current patients/family members, male, ≤ high school education) preferred clinics or hospitals while those who were predicted to access convenient services (i.e., most clinicians, female, education > high school) preferred their home. Lastly, Cunningham et al. (2017) found that university students with lower intent to use face-to-face mental health services (more likely to be men) preferred their home, compared to university students who wanted services with alternative (e.g., exercise, diet) treatments offered (more likely to be women) where a campus student health centre was preference.

Delivery Mode and Format: Eleven studies identified preferences for modes of delivery (Bastien et al., 2021; Batterham & Calear, 2017; Becker et al., 2016; Cunningham et al., 2017; Klein & Cook, 2010; Lau et al., 2012; Lokkerbol et al., 2019a,b; Phillips et al., 2022; Phillips et al., 2021; Tauscher et al., 2023), with most reporting a preference for in-person delivery. Two studies (Lokkerbol et al., 2019a,b) identified a preference for a combination of in-person and tech-enabled delivery. Only one study (Lau et al., 2012) found a preference for tech-enabled delivery (i.e., via telephone) over in-person, in a sub-group of participants who favoured individual therapy. Four studies reported on the format of service delivery, which focused on e-mental health programs (i.e., web-based psychoeducation materials and/or videos) (Batterham & Calear, 2017; Klein & Cook, 2010; Muntingh et al., 2019; Phillips et al., 2021). Two Australian studies found that participants from the general population preferred an informative text format for e-mental health programs (Batterham & Calear, 2017; Klein & Cook, 2010) while one study found that participants recruited from the general population in Germany preferred a video format (Phillips et al., 2021). The fourth study did not find a significant preference for delivery format among Dutch patients with a remitted depressive or anxiety disorder (Muntingh et al., 2019).

Frequency and Timing: Seven studies investigated session frequency (Bastien et al., 2021; Dwight-Johnson et al., 2013; Katz et al., 2020; Lokkerbol et al., 2019a,b; Muntingh et al., 2019; Tauscher et al., 2023), with most finding either no significant preference, or a preference for weekly sessions. Muntingh et al. (2019) found that participants preferred quarterly sessions with a professional, but this was supplemented by a weekly virtual self-guided mental health program. The only study to focus on smoking cessation found veteran outpatients preferred five or more follow-up sessions after an initial session, compared to four or fewer sessions (Katz et al., 2020). Timing of appointments was reported in five studies (Becker et al., 2016, 2019; Hawke et al., 2021a, b; Lau et al., 2012), with mixed preferences (e.g., no appointments needed, weekday afternoons). However, services offering a wider range of appointment times were preferred by participants in two studies (Hawke et al., 2021a, b). Lastly, wait time was included in eight studies (Becker et al., 2016; Cunningham et al., 2017; Hawke et al., 2021a, b; Lau et al., 2012; Lokkerbol et al., 2019a,b; Tauscher et al., 2023), with all except one reporting a preference for no wait time.

Convenience: Six studies reported on convenience of appointments (Dwight-Johnson et al., 2004, 2010, 2013; Hawke et al., 2021a, b; Katz et al., 2020), with most finding a preference for including optional telephone appointments, bus passes, e-health service options and online booking options to make attending appointments easier. Alternatively, Katz et al. (2020) reported a negative preference for certain convenience strategies, such as telephone counselling and unscheduled counselling on request, compared to scheduled, in-person counselling in a Veterans Affairs clinic.

Other: Six studies included features related to extra treatment supports (Batterham & Calear, 2017; Becker et al., 2016, 2019; Dwight-Johnson et al., 2013; Hawke et al., 2021a, b), with all reporting a preference for services that offered treatment for physical or substance use problems in addition to mental health challenges. Service engagement features were included in five studies (Becker et al., 2016, 2019; Cunningham et al., 2017; Hawke et al., 2021a, b), with all reporting a preference for services that educate the community about mental health or involve people with lived experience in the design and feedback process. Cost was included as a feature in four studies (Dwight-Johnson et al., 2010, 2013; Phillips et al., 2021; Tauscher et al., 2023). All studies reported a preference for lower cost services, with the highest preference for free services.

### Treatment Feature Preferences

Nine features were identified that aligned with the theme of treatment features. All studies, except one (Bastien et al., 2021), included at least one of the features in this theme, with four features included in four or more studies.

Treatment Type: Seven studies reported on preferences for treatment type (Becker et al., 2019; Cunningham et al., 2017; Dwight-Johnson et al., 2004, 2010, 2013; Klein & Cook, 2010; Muntingh et al., 2019). Most studies found that participants preferred a combination of counselling and medication or their choice of treatment (i.e., medication, counselling, or both) (Becker et al., 2019; Cunningham et al., 2017; Dwight-Johnson et al., 2004, 2010). Dwight-Johnson et al. (2013) reported that men of Mexican origin preferred counselling whereas White non-Hispanic men preferred medication. In another study (Klein & Cook, 2010), participants who favoured eHealth preferred an internet-based program with therapist-assistance, while those that did not favour eHealth preferred medication. It should be noted that this study did not present traditional in-person counselling as a specific option. Muntingh et al. (2019) was the only study not to find a significant preference for treatment type. However, this study compared four different psychotherapies rather than comparing counselling and medication.

Treatment Format: Six studies reported on treatment format (Becker et al., 2019; Dwight-Johnson et al., 2004, 2010; Lau et al., 2012; Lokkerbol et al., 2019a,b), with the majority finding a preference for individual sessions over group sessions. Becker et al. (2019) reported that one group of participants (mostly clinicians) preferred small group sessions alongside individual sessions, while the other group of participants (mostly patients) preferred individual sessions only. Dwight-Johnson et al. (2010) reported no significant preference for treatment format.

Other: Treatment effectiveness was reported by eight studies, with all finding a preference for more effective treatment (Becker et al., 2016; Cunningham et al., 2017; Katz et al., 2020; Lau et al., 2012; Muntingh et al., 2019; Phillips et al., 2021, 2022; Tauscher et al., 2023). However, two studies reported a preference for services that other people with mental health problems found helpful, over those supported solely by research evidence or clinical opinion (Becker et al., 2016; Cunningham et al., 2017). Four studies (Becker et al., 2019; Hawke et al., 2021a, b; Katz et al., 2020) reported on collaborative decision-making, with all reporting a preference for service providers to work collaboratively with the patient in shaping the treatment plan.

### Clinician Feature Preferences

Five features were identified that aligned with the theme of clinician features. Nine of the 19 studies included at least one of the features in this theme, with two features included in four or more studies.

Professional Background: Five studies reported a preference for a psychologist or psychiatrist, compared to a mental health nurse, social worker, general practitioner, or (peer) counsellor (Becker et al., 2016, 2019; Cunningham et al., 2017; Dwight-Johnson et al., 2013; Klein & Cook, 2010). One study (Katz et al., 2020) reported a preference for a clinical counsellor (e.g., nurse), rather than a non-clinical counsellor (e.g., health coach) or peer counsellor (coaching by another veteran). Participant demographics introduced some variability in preferences across studies. For example, Dwight-Johnson et al. (2013) found that men of Mexican origin preferred a psychiatrist yet identified no significant preference in White non-Hispanic men. Becker et al. (2016) noted that participants who favoured traditional mental health care preferred a psychologist or psychiatrist while participants who favoured convenient mental health care preferred a mental health nurse. A subsequent study by the same team reaffirmed this, finding that one group of participants (mostly mental health professionals) preferred that mental health treatment for service users was provided by a mental health nurse, while another group (mostly patients) preferred that mental health treatment for service users was provided by a psychologist or psychiatrist (Becker et al., 2019). Lastly, Cunningham et al. (2017) found two distinct groups of preferences for provider amongst university students; those that preferred a psychologist or psychiatrist, and those that preferred a peer counsellor. The former group was characterised by hesitation to access in person services, whilst the latter group were characterised by favouring services with alternative treatments (e.g., exercise, diet).

Cultural Background: Six studies identified preferences for the cultural background of clinicians (Becker et al., 2019; Becker et al., 2016; Cunningham et al., 2017; Dwight-Johnson et al., 2013; Hawke et al., 2021a, b), with all but one (Dwight-Johnson et al., 2013) reporting a statistically significant preference. Five studies found a preference for culturally sensitive services or the option of talking to a service provider from their own cultural background. However, two studies identified specific exceptions based on participant sub-groups. Cunningham et al. (2017) found that university students hesitant to access in-person services had no significant preference related to the cultural background of the service provider. Hawke, Thabane, Wilkins, Hawke et al. (2021b) found that caregivers who prioritised a high level of involvement in a young person’s care preferred that the cultural background of service providers not be a determining factor.

### Additional Supports Preferences

Nine features were identified that aligned with the theme of additional supports. Ten of the 19 studies included at least one of the features in this theme, with only one feature included in four or more studies.

Family/Caregiver Involvement: Six studies (Becker et al., 2016, 2019; Dwight-Johnson et al., 2010, 2013; Hawke et al., 2021a, b) reported a preference for involving families/caregivers in the treatment process if the patient chooses.

## Discussion

Virtual care delivery offers many benefits to users of mental health and substance use services; however, it also has many barriers to its use. A user-centred approach which identifies the preferred features of telehealth services can help to ensure that telehealth is implemented in a way that will be used effectively and by all. Although there are specific advantages to telehealth, knowledge about user preferences for virtual care more broadly can help to inform this user-centred approach.

This systematic review sought to identify what factors influenced preferences for virtual service delivery in mental health and substance use settings. We found no studies in which the primary research question was to investigate how different service features influenced preferences for different models of virtual care. However, nineteen studies met our eligibility criteria as they examined some component of virtual care (e.g., included a modality attribute) as part of investigating preferences for other care models (e.g., different models of treatment). Features identified in the studies were grouped into four thematic categories: service features, treatment features, clinician features, and additional supports. Although the aim was to understand preferences for virtual care delivery, to be included in this review studies only had to have at least one feature related to virtual care – they were not required to include, or report on, a direct comparison of in-person care with virtual care. As such, only nine of the 41 features identified were related to virtual care delivery. Most studies focused on treatment features and only included one feature related to virtual delivery, for example, as an alternative to in-person care. These studies typically reported the influence the virtual modality had on overall preferences or likelihood of uptake (e.g., offering telephone appointments increased treatment acceptance), but not how the other features influenced preferences or uptake of virtual care. These studies can thus provide information on the overall preferences for (or against) virtually provided care, but they do not provide information for service providers seeking to design a virtual care service that minimises the specific barriers associated with virtual care delivery and predict its uptake based on different service features. Four studies examined preferences for online mental health programs (Batterham & Calear, 2017; Klein & Cook, 2010; Muntingh et al., 2019; Phillips et al., 2021), but focused on self-help tools without clinician contact. Thus, whilst the preferences for additional e-health supports are covered, the specific features of virtual care services in mental health and substance use settings remain largely unexplored in preference elicitation studies.

Within the theme of service features, most studies reported a preference for in-person services, delivered at home or in a clinical setting once per week with no wait time. While some studies identified a preference for receiving treatment at home, compared to clinical locations, this preference was dependent on demographics factors, such as gender and cultural background. Although one of the benefits of virtual care is its ability to be ‘provided at home’, it is important to note that the finding from this review of a preference for home-based mental health treatment does not necessarily equate to a preference of virtual care because this finding was independent from modality. Lower education levels were associated with less preference for virtual care. Potential reasons for this may be that people with lower levels of education are less aware of digital technology, more hesitant to use health technology, and less likely to have access to digital technology (Lee et al., 2022).

Within the theme of treatment features, most participants preferred a combination of individual counselling and medication (or a choice of the two) with an emphasis on treatment effectiveness. Preference for treatment type was dependant on cultural background, with men of Mexican origin preferring counselling and White men preferring medication in a US-based sample (Dwight-Johnson et al., 2013). Treatment preference and culture have been linked in previous literature, with some studies identifying a lower preference for treating mental illness with medication among certain cultural groups, such as Latin Americans and African Americans (Lee et al., 2021; Vázquez et al., 2021). This suggests that perhaps the dominant medical model does not always fit with different cultures, and therefore, a model of virtual care which strives to replicate traditional face-to-face care might fail to meet specific cultural needs. As such, future implementations and user-centred designs of telehealth should ensure that different cultural groups and their preferences are incorporated into the model of care.

Within the theme of clinician features there was tendency for demographic factors of the service user to influence cultural background and profession preferences about the service provider. There was a preference for treatment to be provided by a psychologist or psychiatrist with the same cultural background as the service user, and different groups of people (e.g., Mexican vs. White men) reported differences in preferences for provider profession. Further, preferences regarding profession were linked to preferences for other features, such that participants who favoured convenient (i.e., telehealth) mental health care preferred a mental health nurse whereas those who liked traditional (e.g., in-person) mental health care preferred a psychologist or psychiatrist (Becker et al., 2016). These findings highlight that choice of provider could be used to ensure equal uptake of telehealth across different groups, however this is limited by the availability and diversity of the providers. Future research could investigate if increased implementation of virtual care across more services can help to address these availability issues, and whether the choice of service provider is sufficiently important to service users to overcome other barriers to virtual care. At the same time, there may be a need to educate providers to be more culturally responsive and educate users of the advantages of accessing more readily available providers (e.g., mental health nurses) whilst waiting for more traditional but in demand providers (e.g., psychiatrists, psychologists).

Within the theme of additional supports, most features were only included in one or two studies each and were therefore excluded from the synthesised results. There is thus scope for future research to explore whether any of these miscellaneous additional supports consistently found to be important. One feature included in several studies was a preference for family/caregiver involvement in treatment planning. A recent systematic review shed light on the benefits and drawbacks of family involvement in mental health treatment (Cameron et al., 2022). Family involvement was found to provide support, give comfort, and help monitor symptoms for people receiving mental health treatment. However, at times patients were excluded from discussions about their mental health, or family members lacked mental health literacy, hindering patient treatment and recovery.

Overall, our findings show that there is no ‘one size fits all’ approach for mental health service design and delivery. We found a preference for in-person care over virtual care delivery, whether it be face-to-face or with a virtual component. However, service user and provider preferences for specific, different *virtual* models of care remain unknown. None of the included studies examined preferences for virtual care in populations where it may be most useful, such as people living in rural areas, due to the burden associated with traveling for face-to-face services. The issues regarding virtual care access in these populations has been explored in other research outside the scope of this review (Mseke et al., 2023). Given the advantages of preference elicitation methods in user-centred design, it is important to implement a preference elicitation study that identifies target population preferences before implementing a virtual care service.

### Limitations and Directions for Future Research

This review, and the studies included in this review, are not without limitations. Most studies conceptualised virtual care as an adjunct (e.g., additional app-based support work), rather than an optional substitute to in-person care due to infection, difficulty travelling etc. Additionally, while the findings help to determine people’s preferences for one modality over another, they provided little information on which features of mental health and substance use services would increase uptake of telehealth care, or to what extent. While many qualitative studies have examined the facilitators and barriers to digital health care, preference elicitation methods such as DCEs allow researchers to determine the trade-offs that people make between different sets of preferred features. The experimental design and statistical analysis can enable conclusions, such as `people will switch from preferring in-person to virtual care when it decreases their wait time for an appointment by one week’. These types of conclusions can be easily utilised in implementation frameworks and feasibility assessments of new services. As such, it is important that future preference elicitation studies are designed and analysed such that they explore the preferences for, and predicted utilisation of, different models of telehealth as a supplement (and where appropriate, substitute) for traditional in-person care.

Almost 80% of the included studies either had a sample that that did not compare to their target population, or the authors did not assess whether they had recruited a representative sample. Most samples were skewed towards women, people with higher education levels, and people from White, English-speaking backgrounds. Consequently, the study findings are not representative. Future preference elicitation studies should aim for more representative, un-biased samples by using targeted recruitment methods, rather than recruiting from the general population online. We were not able to compare preferences between service users and service providers within this review, as we only found four studies on providers. Service users and service providers likely have different preferences, which is important to understand when designing and implementing a service so that both groups accommodated. Our review is limited by not being able to draw any conclusions about these groups’ preferences, so another avenue of future research is to compare these groups more directly.

Lastly, since most of the included studies were DCEs, the results presented in this systematic review may not be indicative of real-world utilisation of these services. Most of the DCEs did not include an ‘opt out’ option (i.e., the ability to choose neither option). Consequently, we do not know whether people would choose the said service option if presented with it in real-life circumstances; a dilemma discussed at length in the DCE literature (Determann et al., 2019; Lancsar & Donaldson, 2005; Quaife et al., 2018). Future DCEs in this area should include an ‘opt out’ or ‘status quo’ option, so that the hypothetical preferences (i.e., the DCE results) are more reflective of real-world preferences, and crucially, utilisation.

## Conclusion

To our knowledge, this is the first systematic review to synthesise preference elicitation studies of mental health and substance use services with a virtual care element. Our results suggest that people are more likely to access a mental health or substance use service when it is convenient, affordable, effective, personalised, and face-to-face. Most studies focused on preferences for different mental health treatment programs, where the programs included some form of virtual element (in person care vs. phone call). No studies directly investigated preferences for, or predicted utilisation of, different modalities of services (telehealth vs. traditional in-person care). Thus, our review highlights the need for future preference elicitation studies to specifically investigate which factors influence people’s decision to access telehealth services in both mental health and substance use settings.

## References

[CR1] Australian Bureau of Statistics (2018). *Mental health*. Australian Bureau of Statistics. https://www.abs.gov.au/statistics/health/mental-health/mental-health/2017-18

[CR2] Baker, C. (2020). *Mental health statistics for England: prevalence, services and funding*. The house of commons library. https://dera.ioe.ac.uk/34934/1/SN06988%20(redacted).pdf

[CR3] Bastien, G., Del Grande, C., Dyachenko, A., Kaczorowski, J., Page, M. G., Brissette, S., Lesperance, F., Dubreucq, S., Hooley, P., & Jutras-Aswad, D. (2021). Preferences for research design and treatment of comorbid depression among patients with an opioid use disorder: A cross-sectional discrete choice experiment. *Drug and Alcohol Dependence*, 226. 10.1016/j.drugalcdep.2021.108857 10.1016/j.drugalcdep.2021.108857 34225223

[CR4] Batterham, P. J., & Calear, A. L. (2017). Preferences for internet-based Mental Health interventions in an adult online sample: Findings from an Online Community Survey. *JMIR Mental Health*, *4*(2). 10.2196/mental.7722 10.2196/mental.7722 PMC5511366 28666976

[CR6] Becker, M. P., Christensen, B. K., Cunningham, C. E., Furimsky, I., Rimas, H., Wilson, F., Jeffs, L., Bieling, P. J., Madsen, V., Chen, Y. Y., Mielko, S., & Zipursky, R. B. (2016). Preferences for Early Intervention Mental Health Services: A discrete-choice conjoint experiment. *Psychiatric Services (Washington, D. C.)*, *67*(2), 184–191. 10.1176/appi.ps.201400306 26369880 10.1176/appi.ps.201400306

[CR5] Becker, M., Cunningham, C. E., Christensen, B. K., Furimsky, I., Rimas, H., Wilson, F., Jeffs, L., Madsen, V., Bieling, P., Chen, Y., Mielko, S., & Zipursky, R. B. (2019). Investigating service features to sustain engagement in early intervention mental health services. *Early Interv Psychiatry*, *13*(2), 241–250. 10.1111/eip.12470 28836377 10.1111/eip.12470

[CR7] Berardi, C., Antonini, M., Jordan, Z., Wechtler, H., Paolucci, F., & Hinwood, M. (2024). Barriers and facilitators to the implementation of digital technologies in mental health systems: A qualitative systematic review to inform a policy framework. *BMC Health Services Research*, *24*(1), 243. 10.1186/s12913-023-10536-1 38408938 10.1186/s12913-023-10536-1 PMC10898174

[CR8] Bidargaddi, N., Schrader, G., Klasnja, P., Licinio, J., & Murphy, S. (2020). Designing m-Health interventions for precision mental health support. *Translational Psychiatry*, *10*(1), 1–8. 32636358 10.1038/s41398-020-00895-2 PMC7341865

[CR9] Butzner, M., & Cuffee, Y. (2021). Telehealth interventions and Outcomes Across Rural Communities in the United States: Narrative Review. *Journal of Medical Internet Research*, *23*(8), e29575. 10.2196/29575 34435965 10.2196/29575 PMC8430850

[CR10] Caffery, L. J., Farjian, M., & Smith, A. C. (2016). Telehealth interventions for reducing waiting lists and waiting times for specialist outpatient services: A scoping review. *Journal of Telemedicine and Telecare*, *22*(8), 504–512. 27686648 10.1177/1357633X16670495

[CR11] Cameron, S. L., Tchernegovski, P., & Maybery, D. (2022). Mental health service users’ experiences and perspectives of family involvement in their care: A systematic literature review. *Journal of Mental Health*, 1–17. 10.1080/09638237.2022.2091760 35808821

[CR13] Clark, M. D., Determann, D., Petrou, S., Moro, D., & de Bekker-Grob, E. W. (2014). Discrete Choice Experiments in Health Economics: A review of the literature. *Pharmacoeconomics*, *32*(9), 883–902. 10.1007/s40273-014-0170-x 25005924 10.1007/s40273-014-0170-x

[CR12] Clark, D. M., Canvin, L., Green, J., Layard, R., Pilling, S., & Janecka, M. (2018). Transparency about the outcomes of mental health services (IAPT approach): An analysis of public data. *The Lancet*, *391*(10121), 679–686. 10.1016/S0140-6736(17)32133-5 10.1016/S0140-6736(17)32133-5 PMC5820411 29224931

[CR14] Coombs, N. C., Meriwether, W. E., Caringi, J., & Newcomer, S. R. (2021). Barriers to healthcare access among U.S. adults with mental health challenges: A population-based study. *SSM - Population Health*, *15*, 100847. 10.1016/j.ssmph.2021.100847 34179332 10.1016/j.ssmph.2021.100847 PMC8214217

[CR15] Cunningham, C. E., Zipursky, R. B., Christensen, B. K., Bieling, P. J., Madsen, V., Rimas, H., Mielko, S., Wilson, F., Furimsky, I., Jeffs, L., & Munn, C. (2017). Modeling the mental health service utilization decisions of university undergraduates: A discrete choice conjoint experiment [Empirical study; quantitative study]. *Journal of American College Health*, *65*(6), 389–399. 10.1080/07448481.2017.1322090 28511031 10.1080/07448481.2017.1322090

[CR16] Dattani, S., Ritchie, H., & Roser, M. (2021). *Mental Health*. https://ourworldindata.org/mental-health

[CR17] Determann, D., Gyrd-Hansen, D., de Wit, G. A., de Bekker-Grob, E. W., Steyerberg, E. W., Lambooij, M. S., & Bjørnskov Pedersen, L. (2019). Designing unforced choice experiments to inform health care decision making: Implications of using opt-out, neither, or status quo alternatives in discrete choice experiments. *Medical Decision Making*, *39*(6), 681–692. 31354031 10.1177/0272989X19862275

[CR18] Dubreucq, J., Plasse, J., & Franck, N. (2021). Self-stigma in serious mental illness: A systematic review of frequency, correlates, and consequences. *Schizophrenia Bulletin*, *47*(5), 1261–1287. https://www.ncbi.nlm.nih.gov/pmc/articles/PMC8563656/pdf/sbaa181.pdf 33459793 10.1093/schbul/sbaa181 PMC8563656

[CR20] Dwight-Johnson, M., Lagomasino, I. T., Aisenberg, E., & Hay, J. (2004). Using Conjoint Analysis to assess Depression Treatment preferences among Low-Income latinos [Empirical study; quantitative study]. *Psychiatric Services*, *55*(8), 934–936. 10.1176/appi.ps.55.8.934 15292545 10.1176/appi.ps.55.8.934

[CR21] Dwight-Johnson, M., Lagomasino, I. T., Hay, J., Zhang, L., Tang, L., Green, J. M., & Duan, N. (2010). Effectiveness of collaborative care in addressing depression treatment preferences among low-income latinos. *Psychiatric Services (Washington D C)*, *61*(11), 1112–1118. 10.1176/ps.2010.61.11.1112 21041350 10.1176/ps.2010.61.11.1112

[CR19] Dwight-Johnson, M., Apesoa-Varano, C., Hay, J., Unutzer, J., & Hinton, L. (2013). Depression treatment preferences of older white and Mexican origin men. *General Hospital Psychiatry*, *35*(1), 59–65. 10.1016/j.genhosppsych.2012.08.003 23141027 10.1016/j.genhosppsych.2012.08.003 PMC4041603

[CR22] Elbogen, E. B., Lanier, M., Wagner, H. R., & Tsai, J. (2021). Financial strain, mental illness, and homelessness: Results from a national longitudinal study. *Medical Care*, *59*, S132–S138. 33710085 10.1097/MLR.0000000000001453

[CR23] Ellis, L. A., Meulenbroeks, I., Churruca, K., Pomare, C., Hatem, S., Harrison, R., Zurynski, Y., & Braithwaite, J. (2021). The application of e-mental health in response to COVID-19: Scoping review and bibliometric analysis. *JMIR Mental Health*, 8(12), e32948. 10.2196/32948 PMC8651237 34666306

[CR24] Greenwood, H., Krzyzaniak, N., Peiris, R., Clark, J., Scott, A. M., Cardona, M., Griffith, R., & Glasziou, P. (2022). Telehealth versus face-to-face psychotherapy for less common mental health conditions: Systematic review and meta-analysis of randomized controlled trials. *JMIR Mental Health*, 9(3), e31780. 10.2196/31780 PMC8956990 35275081

[CR25] Hawke, L. D., Thabane, L., Iyer, S. N., Jaouich, A., Reaume-Zimmer, P., & Henderson, J. (2021a). Service providers endorse integrated services model for youth with mental health and substance use challenges: Findings from a discrete choice experiment. *BMC Health Services Research*, *21*(1), 1–13. 10.1186/s12913-021-07038-3 34598693 10.1186/s12913-021-07038-3 PMC8487137

[CR26] Hawke, L. D., Thabane, L., Wilkins, L., Mathias, S., Iyer, S., & Henderson, J. (2021b). Don’t forget the caregivers! A discrete choice experiment examining caregiver views of Integrated Youth Services. *The Patient*, *14*(6), 791–802. 10.1007/s40271-021-00510-6 33855684 10.1007/s40271-021-00510-6 PMC8046579

[CR27] Hong, Q. N. (2018). *Revision of the mixed methods Appraisal Tool (MMAT): A mixed methods study*. McGill University (Canada).

[CR28] Hughto, J. M., Peterson, L., Perry, N. S., Donoyan, A., Mimiaga, M. J., Nelson, K. M., & Pantalone, D. W. (2021). The provision of counseling to patients receiving medications for opioid use disorder: Telehealth innovations and challenges in the age of COVID-19. *Journal of Substance Abuse Treatment*, *120*, 108163. https://www.ncbi.nlm.nih.gov/pmc/articles/PMC7545305/pdf/main.pdf 33298301 10.1016/j.jsat.2020.108163 PMC7545305

[CR29] Jha, A. K., Ojha, S. P., Dahal, S., Sharma, P., Pant, S. B., Labh, S., Marahatta, K., Shakya, S., Adhikari, R. P., & Joshi, D. (2019). Prevalence of mental disorders in Nepal: Findings from the pilot study. *Journal of Nepal Health Research Council*, *17*(2), 141–147. 31455924 10.33314/jnhrc.v0i0.1960

[CR30] Katz, D. A., Hamlin, C., Vander Weg, M. W., Grant, K. M., Steffensmeier, S., Paez, K. R., Hawley, M., S. T., & Gaeth, G. (2020). Veterans’ preferences for tobacco treatment in primary care: A discrete choice experiment. *Patient Education and Counseling*, *103*(3), 652–660. 10.1016/j.pec.2019.10.002 31629558 10.1016/j.pec.2019.10.002

[CR32] Kim, S., & Tesmer, O. (2021). Employing telehealth strategies for opioid addiction during COVID-19: Implications for social work health care. *Social Work in Health Care*, *60*(6–7), 499–508. 34278979 10.1080/00981389.2021.1953207

[CR31] Kim, H. M., Xu, Y., & Wang, Y. (2022). Overcoming the Mental Health Stigma Through m-Health Apps: Results from the Healthy Minds Study. *Telemedicine and e-Health*. 10.1089/tmj.2021.0418 35254886

[CR33] Klein, B., & Cook, S. (2010). Preferences for e-mental health services amongst an online Australian sample. *E-Journal of Applied Psychology*, *6*(1), 28–39. 10.7790/ejap.v6i1.184

[CR34] Krzyzaniak, N., Greenwood, H., Scott, A. M., Peiris, R., Cardona, M., Clark, J., & Glasziou, P. (2021). The effectiveness of telehealth versus face-to face interventions for anxiety disorders: A systematic review and meta-analysis. *Journal of Telemedicine and Telecare*, 1357633X211053738. 10.1177/1357633X211053738 34860613

[CR35] Lancsar, E., & Donaldson, C. (2005). Discrete choice experiments in health economics. *The European Journal of Health Economics*, *6*(4), 314–316. 10.1007/s10198-005-0304-3 16133096 10.1007/s10198-005-0304-3

[CR36] Lau, M. A., Colley, L., Willett, B. R., & Lynd, L. D. (2012). Employee’s preferences for access to mindfulness-based cognitive therapy to reduce the risk of depressive relapse-A discrete choice experiment [Empirical study; quantitative study]. *Mindfulness*, *3*(4), 318–326. 10.1007/s12671-012-0108-3

[CR38] Lee, M., Lu, W., Mann-Barnes, T., Nam, J. H., Nelson, J., & Ma, G. X. (2021). Mental health screening needs and preference in treatment types and providers in African American and Asian American older adults. *Brain Sciences*, *11*(5), 597. https://mdpi-res.com/d_attachment/brainsci/brainsci-11-00597/article_deploy/brainsci-11-00597.pdf?version=1620207807 34063018 10.3390/brainsci11050597 PMC8148007

[CR37] Lee, C. M. Y., Thomas, E., Norman, R., Wells, L., Shaw, T., Nesbitt, J., Frean, I., Baxby, L., Bennett, S., & Robinson, S. (2022). Educational attainment and willingness to use technology for health and to share health information – the reimagining healthcare survey. *International Journal of Medical Informatics*, *164*, 104803. 10.1016/j.ijmedinf.2022.104803 35644052 10.1016/j.ijmedinf.2022.104803 PMC9760096

[CR39] Lokkerbol, J., Geomini, A., van Voorthuijsen, J., van Straten, A., Tiemens, B., Smit, F., Risseeuw, A., & Hiligsmann, M. (2019a). A discrete-choice experiment to assess treatment modality preferences of patients with depression. *Journal of Medical Economics*, *22*(2), 178–186. 10.1080/13696998.2018.1555404 30501437 10.1080/13696998.2018.1555404

[CR40] Lokkerbol, J., van Voorthuijsen, J. M., Geomini, A., Tiemens, B., van Straten, A., Smit, F., Risseeuw, A., van Balkom, A., & Hiligsmann, M. (2019b). A discrete-choice experiment to assess treatment modality preferences of patients with anxiety disorder. *Journal of Medical Economics*, *22*(2), 169–177. 10.1080/13696998.2018.1555403 30501135 10.1080/13696998.2018.1555403

[CR41] Mahmoud, H., Naal, H., & Cerda, S. (2021). Planning and implementing Telepsychiatry in a community Mental Health setting: A Case Study Report. *Community Mental Health Journal*, *57*(1), 35–41. 10.1007/s10597-020-00709-1 32897476 10.1007/s10597-020-00709-1 PMC7477735

[CR42] Moroz, N., Moroz, I., & D’Angelo, M. S. (2020). Mental health services in Canada: Barriers and cost-effective solutions to increase access. *Healthcare Management Forum*, *33*(6), 282–287. 10.1177/0840470420933911 32613867 10.1177/0840470420933911

[CR43] Mseke, E. P., Jessup, B., & Barnett, T. (2023). A systematic review of the preferences of rural and remote youth for mental health service access: Telehealth versus face-to‐face consultation. *Australian Journal of Rural Health*. 10.1111/ajr.12961 36606417

[CR44] Muntingh, A. D., Hoogendoorn, A. W., Van Schaik, D. J., Van Straten, A., Stolk, E. A., Van Balkom, A. J., & Batelaan, N. M. (2019). Patient preferences for a guided self-help programme to prevent relapse in anxiety or depression: A discrete choice experiment [Empirical Study; Interview; Focus Group; Quantitative Study]. *PLoS ONE Vol 14(7), 2019, ArtID e0219588, 14*(7). 10.1371/journal.pone.0219588 10.1371/journal.pone.0219588 PMC6638925 31318918

[CR45] Naal, H., Mahmoud, H., & Whaibeh, E. (2021). The potential of telemental health in improving access to mental health services in Lebanon: Analysis of barriers, opportunities, and recommendations. *International Journal of Mental Health*, *50*(3), 218–233.

[CR46] Nakao, M., Shirotsuki, K., & Sugaya, N. (2021). Cognitive–behavioral therapy for management of mental health and stress-related disorders: Recent advances in techniques and technologies. *BioPsychoSocial Medicine*, *15*(1), 16. 10.1186/s13030-021-00219-w 34602086 10.1186/s13030-021-00219-w PMC8489050

[CR47] Philip, A., Ford, M., & Goldberg, J. (2022). Getting Beyond Parity: Telehealth as a Best Practice in Health Equity. *Telehealth and Medicine Today*.

[CR49] Phillips, E. A., Himmler, S. F., & Schreyögg, J. (2021). Preferences for e-Mental Health interventions in Germany: A Discrete Choice Experiment. *Value In Health : The Journal of the International Society for Pharmacoeconomics and Outcomes Research*, *24*(3), 421–430. 10.1016/j.jval.2020.09.018 33641777 10.1016/j.jval.2020.09.018

[CR48] Phillips, E. A., Himmler, S., & Schreyögg, J. (2022). Preferences of psychotherapists for blended care in Germany: A discrete choice experiment. *Bmc Psychiatry*, *22*, 1–12. 10.1186/s12888-022-03765-x 35151294 10.1186/s12888-022-03765-x PMC8841060

[CR50] Pluye, P., Gagnon, M. P., Griffiths, F., & Johnson-Lafleur, J. (2009). A scoring system for appraising mixed methods research, and concomitantly appraising qualitative, quantitative and mixed methods primary studies in mixed studies reviews. *International Journal of Nursing Studies*, *46*(4), 529–546. 19233357 10.1016/j.ijnurstu.2009.01.009

[CR51] Polinski, J. M., Barker, T., Gagliano, N., Sussman, A., Brennan, T. A., & Shrank, W. H. (2016). Patients’ satisfaction with and preference for Telehealth visits. *Journal of General Internal Medicine*, *31*(3), 269–275. 10.1007/s11606-015-3489-x 26269131 10.1007/s11606-015-3489-x PMC4762824

[CR52] Quaife, M., Terris-Prestholt, F., Di Tanna, G. L., & Vickerman, P. (2018). How well do discrete choice experiments predict health choices? A systematic review and meta-analysis of external validity. *The European Journal of Health Economics*, *19*(8), 1053–1066. 10.1007/s10198-018-0954-6 29380229 10.1007/s10198-018-0954-6

[CR53] Reichert, A., & Jacobs, R. (2018). The impact of waiting time on patient outcomes: Evidence from early intervention in psychosis services in E ngland. *Health Economics*, *27*(11), 1772–1787. https://www.ncbi.nlm.nih.gov/pmc/articles/PMC6221005/pdf/HEC-27-1772.pdf 30014544 10.1002/hec.3800 PMC6221005

[CR54] Robson, D., & Gray, R. (2007). Serious mental illness and physical health problems: A discussion paper. *International journal of nursing studies, 44*(3), 457–466. 10.1016/j.ijnurstu.2006.07.013 10.1016/j.ijnurstu.2006.07.013 17007859

[CR55] Ryan, M., Gerard, K., & Amaya-Amaya, M. (Eds.). (2008). *Using discrete choice experiments to value health and health care*. Springer.

[CR56] Scott, A. M., Bakhit, M., Greenwood, H., Cardona, M., Clark, J., Krzyzaniak, N., Peiris, R., & Glasziou, P. (2022a). Real-time telehealth versus face-to-face management for patients with PTSD in primary care: A systematic review and meta-analysis. *The Journal of Clinical Psychiatry*, *83*(4), 41146. 10.4088/JCP.21r14143 35617629

[CR57] Scott, A. M., Clark, J., Greenwood, H., Krzyzaniak, N., Cardona, M., Peiris, R., Sims, R., & Glasziou, P. (2022b). Telehealth v. face-to-face provision of care to patients with depression: A systematic review and meta-analysis. *Psychological Medicine*, *1*, 9. 10.1017/S0033291722002331 PMC9693715 35959559

[CR58] Smith, J., Kyle, R. G., Daniel, B., & Hubbard, G. (2018). Patterns of referral and waiting times for specialist Child and Adolescent Mental Health Services. *Child and Adolescent Mental Health*, *23*(1), 41–49. 32677372 10.1111/camh.12207

[CR59] Soekhai, V., Whichello, C., Levitan, B., Veldwijk, J., Pinto, C. A., Donkers, B., Huys, I., van Overbeeke, E., Juhaeri, J., & de Bekker-Grob, E. W. (2019). Methods for exploring and eliciting patient preferences in the medical product lifecycle: A literature review. *Drug Discovery Today*, *24*(7), 1324–1331. 10.1016/j.drudis.2019.05.001 31077814 10.1016/j.drudis.2019.05.001

[CR60] Sorkin, D. H., Janio, E. A., Eikey, E. V., Schneider, M., Davis, K., Schueller, S. M., Stadnick, N. A., Zheng, K., Neary, M., & Safani, D. (2021). Rise in use of digital mental health tools and technologies in the United States during the COVID-19 pandemic: Survey study. *Journal of Medical Internet Research*, 23(4), e26994. 10.2196/26994 PMC8054774 33822737

[CR61] Stagnaro, J. C., Cía, A. H., Gaxiola, A., Vázquez, S., Sustas, N., Benjet, S., C., & Kessler, R. C. (2018). Twelve-month prevalence rates of mental disorders and service use in the Argentinean Study of Mental Health Epidemiology. *Social Psychiatry and Psychiatric Epidemiology*, *53*(2), 121–129. 10.1007/s00127-017-1475-9 29302708 10.1007/s00127-017-1475-9

[CR62] Tauscher, J. S., DePue, M. K., Swank, J., & Salloum, R. G. (2023). Determinants of preference for telehealth versus in-person treatment for substance use disorders: A discrete choice experiment. *Journal of Substance use and Addiction Treatment*, *146*, 208938. 10.1016/j.josat.2022.208938 36880898 10.1016/j.josat.2022.208938

[CR63] The EndNote Team (2013). *EndNote.* In (Version EndNote X9) [64 bit]. Clarivate.

[CR64] Uscher-Pines, L., Raja, P., Qureshi, N., Huskamp, H. A., Busch, A. B., & Mehrotra, A. (2020). Use of tele–mental health in conjunction with in-person care: A qualitative exploration of implementation models. *Psychiatric Services*, *71*(5), 419–426. https://www.ncbi.nlm.nih.gov/pmc/articles/PMC7271813/pdf/nihms-1573309.pdf 31996115 10.1176/appi.ps.201900386 PMC7271813

[CR65] Valentine, A. Z., Hall, S. S., Young, E., Brown, B. J., Groom, M. J., Hollis, C., & Hall, C. L. (2021). Implementation of Telehealth Services to Assess, Monitor, and treat Neurodevelopmental disorders: Systematic review. *Journal of Medical Internet Research*, *23*(1), e22619. 10.2196/22619 33326409 10.2196/22619 PMC7819544

[CR66] van Kessel, R., Kyriopoulos, I., Wong, B. L. H., & Mossialos, E. (2022). Has the pandemic enhanced and sustained digital health-seeking behaviour? A big data interrupted time-series analysis of Google Trends. *medRxiv*, 2022.2007. 2029.22278191. 10.2196/42401 PMC9848442 36603152

[CR67] Vázquez, A. L., Alvarez, M. C., Flores, N., González, C. M., Vera, J. M., Barrett, T. S., & Domenech Rodríguez, M. M. (2021). Youth mental health service preferences and utilization patterns among Latinx caregivers. *Children and Youth Services Review*, *131*, 106258. 10.1016/j.childyouth.2021.106258

[CR68] Veritas Health Innovation (2021). *Covidence Systematic Review Software.* In Veritas Health Innovation. www.covidence.org.

[CR69] Walker, E. R., McGee, R. E., & Druss, B. G. (2015). Mortality in Mental disorders and Global Disease Burden implications: A systematic review and Meta-analysis. *JAMA Psychiatry*, *72*(4), 334–341. 10.1001/jamapsychiatry.2014.2502 25671328 10.1001/jamapsychiatry.2014.2502 PMC4461039

[CR70] Zhu, D., Paige, S. R., Slone, H., Gutierrez, A., Lutzky, C., Hedriana, H., Barrera, J. F., Ong, T., & Bunnell, B. E. (2021). Exploring telemental health practice before, during, and after the COVID-19 pandemic. *Journal of Telemedicine and Telecare*, *0*(0), 1357633X211025943. 10.1177/1357633x211025943 10.1177/1357633X211025943 PMC10375824 34241545

